# Cervical spine spondylodiscitis: Review of literature on current treatment strategies

**DOI:** 10.1016/j.heliyon.2023.e17875

**Published:** 2023-07-02

**Authors:** Randall W. Treffy, Brandon Laing, Akram M. Eraky, Saman Shabani

**Affiliations:** Department of Neurosurgery, Medical College of Wisconsin, 8701 Watertown Plank Rd, Milwaukee, WI 53226, USA

**Keywords:** Graft, Infection, Instrumentation, Spinal deformity

## Abstract

Infections of the spine are an ever-increasing health concern requiring an often complex and prolonged treatment that can lead to significant morbidity. Of particular interest is the cervical spine where there is an increase rate of post-infectious deformity, secondary neurological deficits and substantially higher rates of associated morbidity and mortality than the thoracic or lumbar spine. In this review, we explore the diagnosis and treatment of spondylodiscitis with particular focus on the cervical spine.

## 1 Introduction

Infections of the spine can lead to significant morbidity as infections can lead to either direct compression of neural elements or indirect compression secondary to development of deformity and resulting in neurological deficits. Infections of the spine can also be difficult to diagnose and can present with subtle and often missed signs on initial presentation. In this review, we will explore the initial presenting symptoms, the diagnostic tests used to identify spondylodiscitis, the causative organisms, and the treatment of spondylodiscitis. We pay particular attention to the cervical spine as infections of the cervical spine can lead to rapid and profound neurological deficits if not appropriately and rapidly treated.

## 2 Presentation

Infection of the spine is a major health concern and has had continued increasing incidence. For example, in one population based study, the incidence of spondylodiscitis was 2.2 cases per 100,000 person years in 1995 and was up to 5.8 cases per 100,000 per years in 2008 [1] while in another population study the incidence was estimated to be 1.47 cases per 100,000 person years from 1995 to 1999 and was up to 3.67 cases per 100,000 person years from 2008 to 2011 [2]. Data for the United States is not as detailed, but in one study focusing on utilization of healthcare resources, the incidence of vertebral osteomyelitis was found to be 2.9 cases per 100,000 per years in 1998 and up to 5.4 cases per 100,000 per years in 2013 [3]. In the first population based study, 9.4% of the cases involved the cervical spine [1] while approximately 7.5% of cases involved the cervical spine in the other population based study [2].

Patient presentation is quite variable, but the most common presenting symptoms are pain (95.5%), fever (58.8%), neurological deficit (21.2%), and weight loss (17.6%) [1]. Other studies support these findings with a meta-analysis of spondylodiscitis presentations demonstrating pain in 91% of cases, fever in 35% of cases, and neurological deficits in 29% of cases [4]. Likely due to the vague complaints, the average time from symptom initiation to diagnosis was approximately 55 days [4].

Associated medical co-morbidities and risk factors found in patients with spondylodiscitis were concurrent infection in 35% of cases, cardiovascular disease in 24% of cases, diabetes in 20% of cases, intravenous drug use in 15% of cases, and immunosuppression in 11% of cases [4]. Alcohol abuse was a contributing comorbidity in 19.2% of cases [1] as well as concurrent malignancy as a common medical comorbidity in 11% of cases [2]. The average age of presentation was found to be 63.3 and 66.6 in two population studies, while it was 58.3 in a meta-analysis [1,2,4].

## 3 Diagnosis

Diagnosis of spondylodiscitis is a combination of laboratory results as well as imaging results in a patient with the appropriate history.

### 3.1 Laboratory tests

C-reactive protein (CRP) is an acute phase reactant and its serum level can rise nearly 1000 fold in active infection [5]. Laboratory measurement of CRP is a useful diagnostic tool to assist with diagnosis of infection, including within the spine, and the level will often normalize with treatment [[6], [7], [8]]. Another laboratory test that can be used to monitor infection is the erythrocyte sedimentation rate (ESR), which measures erythrocyte rouleaux formation, an indirect measurement of inflammatory processes [9]. ESR is also found to be elevated in spondylodiscitis [[7], [8]]. Utilizing CRP and ESR together often increases the diagnostic utility when looking for an acute infection in general [9] and can be used together to diagnose spondylodiscitis [10]. However, CRP appears to respond more rapidly to infection and normalize more quickly than ESR and often is the choice to track active infection and treatment [8].

Another laboratory test that has been examined is procalcitonin (PCT). PCT is a 116-amino acid peptide, which is typically cleaved into calcitonin to assist with calcium homeostasis [11]. When bacterial endotoxins are present, or with elevated levels of cytokines, the level of serum PCT can raise 100–1000 fold [11]. It does not have as well of an established role in spondylodiscitis, but it was found to be less sensitive than CRP and is only significantly elevated in patients with concurrent spondylodiscitis and other concurrent infections [12]. As more studies are done looking at PCT, it may potentially become more useful in helping determine if patient's have concurrent infectious as their etiology of spondylodiscitis instead of simply localized disease.

### 3.2 Imaging

Although laboratory tests can detect active infection, in order to localize the infection, diagnostic imaging should be obtained. Classically radiographs are the easiest and quickest imaging modality, but radiographs have a reported specificity of 57%, sensitivity of 82%, and accuracy of 73% and often take 2–8 weeks to show changes on imaging; these changes often must be quite significant to be appreciated [[13], [14], [15]]. Advanced neuroimaging is more sensitive and specific than plain radiographs and has become standard.

Magnetic resonance imaging (MRI) has a specificity of 94%, sensitivity of 96%, and accuracy of 92% and is thought to be the imaging modality of choice for diagnosing infections of the spine [14,16,17]. Expected imaging changes are decreased T1 intensity from involved areas, increased T2-weighted intensity, and gadolinium enhancement of the infected disc, vertebrae, and surrounding soft tissues [13,14,16,17] Although MRI is the choice of imaging for diagnosis of spinal infections, computed tomography (CT) can be performed to better elucidate bone anatomy; which can assist in determining if surgical intervention is indicated [14,18,19]. CT scans are also often used for guidance for needle biopsies, if indicated [14].

Bone scintigraphy, which usually uses technetium-99m-methylene bisphosphonate, which binds to hydroxyapatite and in theory would be increased in osteomyelitis due to local hyper perfusion, has a sensitivity of 36% and specificity of 92% for an accuracy of 67% [20]. Because of its low sensitivity and specificity, bone scintigraphy is typically not used as a sole imaging modality [21,22]. Gallium-67 is a radioactive atom that binds to transferrin and therefore will locally accumulate in areas of increased perfusion and vascular permeability, such as areas of active infection [22]. When used with bone scintigraphy, it increases diagnostic accuracy (85% specific, 64% sensitive, and 92% accurate), and when done using single photon-tomography it is equally accurate alone as with bone scintigraphy (92% specific, 91% sensitive, 92% accurate) [20]. Another radiotracer is indium-111 tagged to biotin; which is a molecule not typically found in the spine but is important for bacterial cell growth [22]. When used in a limited study to examine spinal infections, indium-111 tagged to biotin was 95% specific and 94% sensitive [23]. In a larger study, the same group found a specificity of 98%, sensitivity of 84%, and accuracy of 92% in hematogenous osteomyelitis while they found a specificity of 84%, sensitivity of 100%, and accuracy of 92% in suspected surgical site spinal infection [24]. Although this imaging modality appears promising, there have not been other studies that further examined its feasibility.

The most commonly utilized nuclear medicine imaging modality for investigating spinal infections is typically thought of as FDG-PET [22,25]. FDG can be transported into cells via glucose transporters, once in the cell it is phosphorylated via hexokinase at which point it can both no longer exit the cell and can no longer be metabolized further [25]. It is known to accumulate within leukocytes at the source of infection as leukocytes at these locations typically have elevated metabolic rates [25]. There are numerous studies indicating the high diagnostic utility of FDG-PET imaging [22]. Of particular interest, in one study looking at endplate abnormalities in 30 patients, FDG-PET was both 100% sensitive and specific for underlying infectious process while MRI was 50% sensitive and 96% specific [26]. In another study of 38 patients with inconclusive MRI findings, FDG-PET had a sensitivity of 81.8%, specificity of 100%, and accuracy of 89.5% while conventional MRI had a sensitivity of 75%, specificity of 71.4%, and accuracy of 74.1% [27]. A somewhat recent meta-analysis further examined 396 patients from 12 studies and found an overall sensitivity of 94.8% and specificity of 91.4%, further supporting the utility of FDG-PET imaging [28]. These findings suggest that FDG-PET can be a useful tool in the armament of neurosurgeons to further identify areas of infection in cases with otherwise inconclusive imaging.

### 3.3 Cultures

To appropriate treat spondylodiscitis (see below), the appropriate causative organism should be determined. The vast majority of the time, the cause of the infection is a single organism; but polymicrobial infections do occur in the setting of chronic infections [29]. It is best to obtain cultures prior to initiation of antibiotics to identify the causative agent if the patient is not septic [30]. If definitive causative agent cannot be safely obtained prior to starting antibiotics, blood cultures should at a minimum be obtained prior to antibiotic therapy and can be positive in up to 70% of patients not already receiving antibiotics [31]. Of note, in a single center study, blood cultures increased definitive diagnosis significantly over only intraoperative cultures (66.3%–80.6%) [32]. The most common causative organism is *Staphylococcus aureus* (15–84%), followed by gram-negative bacilli (typically E*scherichia coli, Pseudomonas aeruginosa,* and P*roteas mirabilis* [33]) (4–30%), then S*treptococci* species and *Enterococcus* species (5–30%) [29]. Interestingly, methicillin-sensitive *Staphylococcus aureus* was twice as likely to be associated with cervical spondylodiscitis while no other bacteria had a predilection for the cervical spine [32]. Once the causative organism is identified, antibiotic therapy can be a tailored to that organism.

#### 3.3.1 Nonoperative management

Both operative and nonoperative management may be viable options for cervical spondylodiscitis depending on the patient's clinical presentation. Decision-making in these patients can be difficult as clinicians must factor in both the risk of present and potential delayed spinal instability, cervical deformity, neurologic status, and infection control.

Nonoperative management is primarily reserved for patients with early spondylodiscitis, lack of neurologic deficits, and minimal bony disruption. In these cases, prior literature suggests that conservative treatment with rigid orthosis and antibiotic administration for at least 6 weeks is reasonable [34]. However, prior to initiation of antibiotics, an infectious organism should be identified if possible. In the absence of a known infectious organism, image guided needle biopsy is recommended prior to antibiotic administration unless there is evidence of impending sepsis or hemodynamic instability [35]. Reports from a systemic review and meta-analysis suggests up to 50% of biopsies do not yield a causative organism [36], in those situations blood cultures (if positive) can guide antibiotic choice otherwise antibiotics will have to be empiric only [35]. A systematic review of second percutaneous image-guided biopsy after initial negative biopsy found extremely variable results (0–60% diagnostic yield), therefore evidence for a second biopsy after an initial negative biopsy is limited and is not routinely advised [37].

##### 3.3.1.1 Antibiotic management

Long-term antibiotic therapy is required for control of cervical spondylodiscitis. Antibiotic treatment consists primarily of at least 6 weeks of antibiotic therapy. Choice of antibiotic largely depends on the organism. To treat gram-positive infections, typically a semi-synthetic penicillin such as oxacillin or nafcillin, or first or second generation cephalosporins can be used. For methicillin-resistant gram-positive bacteria, vancomycin, teicoplanin, and linezolid are valid options. To treat gram-negative bacteria, antibacterial coverage is differentiated based on pseudomonas coverage. For anti-pseudomonal coverage, beta lactams such as ticarcillin, piperacillin-tazobactam, cefepime, ceftazidime, or carbapenems may be applicable. For non-pseudomonal, gram-negative coverage, second or third generation cephalosporins, quinolones, or aminoglycosides may be used [38].

##### 3.3.1.2 Radiographic surveillance

The role of radiographic surveillance in therapy response is controversial. As previously mentioned, MRI imaging is the modality of choice for initially evaluating spondylodiscitis given its high sensitivity. However, post-treatment, MRI imaging may show as persistent and potentially worsening enhancement despite improvements in clinical status and infection control [39]. Because of this discrepancy, routine follow-up MRI for radiographic surveillance is not recommended. Instead, inflammatory markers such ESR and or CRP should be completed after 4 weeks of antimicrobial therapy [35].

#### 3.3.2 Surgical management

Surgical management is commonly indicated in cases with advanced spondylodiscitis, evidence of neurologic deficit or spinal instability, new or worsening kyphosis, failure of conservative management, need for infectious source control, and or lack of a diagnostic organism. Evidence of neurologic comprise with or without impending sepsis has been deemed a strong indication for surgery and empirical antibiotics. Commonly, the goal of surgical intervention is radical debridement of infected tissue, spinal stabilization, and neural element decompression. There is evidence that surgical intervention may be more readily indicated in cases of cervical spondylodiscitis compared to thoracolumbar. Compared to thoracolumbar spondylodiscitis, cervical infections have been associated with a high morbidity and mortality rate. For example, in a cohort of 15 cases of cervical spondylodiscitis, 67% of patients presented with a neurologic deficit at time of diagnosis [40] while a study of 19 patients at another center had similar findings [41]. Due to this high incidence of neurologic deficits, some authors suggest that early surgical treatment should be considered for patients with cervical spondylodiscitis and lack of surgical contraindications [42]. In one metanalysis in which 12% of cases (459) involved the cervical spine, surgical outcomes were associated with improved pain scores, but the heterogenous nature of the data as well as the combination of cervical, thoracic, and lumbar into a single data pool make this difficult to interpret [4]. Even in patients who have significant neurological deficits, significant recovery can occur with prompt surgical intervention, as demonstrated by a literature review in which 1/5 of patients with a complete neurological injury due to cervical or thoracic spondylodiscitis/epidural abscess were ambulatory at follow up [43]. Interestingly, a single case study demonstrated that prompt antibiotic management alone improved a patient's neurological status with cervical spondylodiscitis/epidural abscess [44].

##### 3.3.2.1 Anterior vs. posterior

Surgical treatment can consist of anterior, posterior, or combined circumferential decompression with or without instrumentation. There is no clear consensus on surgical approach, however anterior cervical debridement is more commonly cited in the literature. Furthermore, there is evidence that anterior cervical decompression has been associated with improved motor function and a greater degree of neurologic benefit compared to anterior-posterior treatments [45]. Although the data in the previous mentioned study may be subjected to bias based on clinical presentation; it is thought that potentially kyphosis and ventral cord compression is likely worse with ventral infections compared to dorsal infections potentially leading to quicker surgical intervention. Although earlier studies have also demonstrated good results with anterior intervention only with anterior-posterior approaches saved for those that fail anterior only [46]. With regards to combined-anterior-posterior approaches, circumferential fixation is recommended when anterior cervical corpectomies are needed or if there is multisegmental disease involvement [42,47]. Larger case series comparing surgical approaches would be helpful to further analyze the best treatment paradigm. For now, it appears the best approach would be to surgically decompress the lesion directly and stabilize through that same approach; for multisegmental involvement an anterior-posterior approach to further provide stability and prevent further deformity appears most appropriate.

##### 3.3.2.2 Instrumentation

The role and risks of instrumentation in cervical spondylodiscitis have been debated previously. Prior studies have shown that there is a risk of asymptomatic bacterial colonization even one year out of surgery with anterior cervical plating [48] and previous recommendations were to remove spinal hardware [49], which likely led to significant worsening deformity [50]. However, Shad et al. was a small case series of five patients and these patients were largely asymptomatic [48]. Furthermore, there are various authors that have documented safe outcomes with instrumentation in the setting of spondylodiscitis [51,52]. It is important to consider the risk of subsidence with implantation of an inter-body cage in the setting of infection. In a study comparing polyetheretherketone (PEEK) and titanium implants in the setting of spondylodiscitis, Schomacher et al. found that they had a subsidence rate of around 70% with either PEEK or titanium implants [53]. This high rate of subsidence is theorized to be due to the poor bone quality and disruption of the vertebral endplates due to the chronic inflammation of the vertebrae in cases of advanced spondylodiscitis. However, this endplate disruption also likely improves the fusion rates. In this same cohort, the rate of fusion 90.5% and 100% in the PEEK and titanium groups respectively without any reinfections [53]. This study as well as a study focusing on the lumbar spine found no significant difference in fusion or subsidence rates in the setting of infection when using PEEK vs titanium cages [53,54]. Given these findings, no suggestion of PEEK vs titanium cages can be made, and the decision should be made by the surgeon based on comfort and availability.

Fusion and deformity outcomes appear to be better in patients with instrumentation compared to those without. In a multi-center trial comparing outcomes of anterior cervical debridement and fusion with or without cervical plating, cervical instrumentation led to better fusion rates, improved segmental height, and better segmental and C2–C7 angles [55]. In addition, there was a 0% chance of infection recurrence in both groups.

In summary, it appears that it is generally safe to place titanium or PEEK hardware in the setting of a cervical spine infection as the rate of fusion in the above studies is higher with hardware and the rate of recurrent infection is low.

##### 3.3.2.3 Grafts

In the setting of spondylodiscitis, there may be concern for using grafting material as a nidus of infection. However, in the setting of surgical cervical spondylodiscitis, multiple groups advocate for the use of iliac crest grafting after debridement of the infected tissue without significant recurrence of the infection [56,57]. Classically, allografts were avoided as well as it was thought that allografts had a higher rate of recurrence. However, allografts and autografts have similar fusion rates and similar low recurrence rates (around 5%) [58,59], suggesting one can use autograft, if possible, but if autograft cannot be obtained, allograft can be safely used. Although data comparing allografts directly is sparse; it appears that cadaveric bone grafts [60,61] as well as bone chips with demineralized bone-matrix [62] are effective treatment strategies. BMP has also been used with good fusion rates and without associated re-infection [63]. Further head-to-head comparison data would be beneficial to determine the optimal treatment paradigm for spinal infections, but at this time, there appears to be no significant difference with grafting material as long as appropriate instrumentation has been performed.

#### 3.3.3 Treatment failure

The rate of treatment failure between conservative and surgical treatment has been discussed in the literature. In Valancius et al.‘s study of patients with spondylodiscitis, they found that 13.1% of patients had failure of conservative management either due to persistent or worsening infection or development of new neurologic deficits [64]. Contrastingly, every patient in the surgery group had control of their infection postoperatively, without any recurrence or persistent infection despite placement of foreign hardware. Of the seven patients in their cohort surgically treated for cervical lesions, only one patient required re-operation due to a postoperative hematoma [64]. It is important to note that this rate of failure for conservative management is across all forms of spondylodiscitis, which primarily affects the lumbar spine. In addition, Valancius et al.‘s study was composed of only 9 patients with cervical discitis, the majority of whom required surgical intervention [64]. Another case series and meta-analysis of all locations of spondylodiscitis again found low rates of treatment failure with conservative management in their own case series as well as meta-analysis [65], but they did find that patients with cervical spondylodiscitis had an odd's ratio of 4.03 for need for surgical intervention and odd's ratio of 4.16 for neurological deficits; further supporting the evidence that spondylodiscitis of the cervical spine has a higher rate of morbidity compared to that of the thoracic and lumbar spine [65]. There is sparse literature specifically looking at failure of conservative management specifically with cervical spondylodiscitis. More specific studies need to be performed focusing on conservative management of cervical spondylodiscitis to further determine if there are differences in treatment failure for the cervical spine compared to much more common thoracic and lumbar spondylodiscitis.

#### 3.3.4 Non-pyogenic causes of spondylodiscitis

Although rare in the United States and Western Europe, non-pyogenic causes of spondylodiscitis are common throughout other areas of the world [51,66]. Tuberculosis associated spondylodiscitis (Pott's disease) represents approximately 46% of all spondylodiscitis cases in the world and is a major cause of morbidity and mortality with a large percentage of patients presenting with neurological deficits [51,66]. In a retrospective case review of 694 patient's treated for Pott's disease in Turkey, approximately 4.2% were found to have lesions of the cervical spine (55.8% thoracic, 22.8% lumbar, 16.9% thoracolumbar) [66]. Diagnostic techniques were similar to pyogenic causes utilizing advanced imaging techniques and cultures but nearly all of the cases were treated surgically with decompressions and fusion (682; 98.2%) followed by treatment with rifampin, isoniazid, pyrazinamide, and ethambutol/streptomycin for 2–6 months followed by further suppressive therapy for 6–18 months [66].

Another non-pyogenic cause of spondylodiscitis rare in the United States and Western Europe is brucellosis. Another case series examined 452 cases of spinal brucellosis and found 21 (4.6%) cases involving the cervical spine [67]. Interestingly, brucellosis spondylodiscitis was rarely treated surgically (17%; and no fusions) and instead the patients were mostly treated with a combination of antibiotics including rifampicin, doxycycline, streptomycin, tetracycline, and ofloxacin [67].

Although rare in the United States and Western Europe, non-pyrogenic causes of spondylodiscitis are fairly common in the rest of the world. At this time, there is little data specifically regarding the cervical spine, but one can presume from the data on pyrogenic spondylodiscitis that involvement of the cervical spine also likely leads to significant associated morbidity and mortality and special attention should be paid to these infections organisms in the cervical spine.

#### 3.3.5 Case examples

##### 3.3.5.1 Case 1

**Fig. 1:**
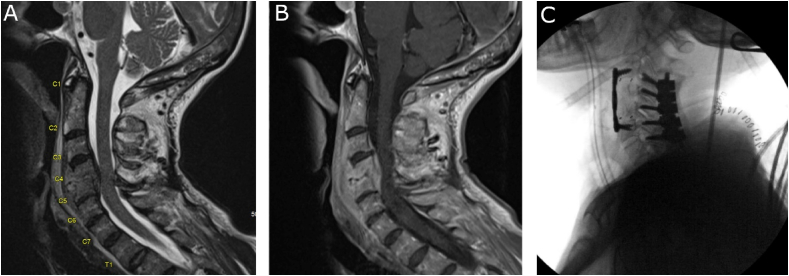
Example of a case of cervical spondylodiscitis with associated epidural abscess treated at our center. A: Sagittal T2 image demonstrating significant canal stenosis with developing kyphotic deformity centered at C4/5. B: Sagittal T1 post-contrast image demonstrating contrast enhancement within the disc and vertebral bodies of C4/5 with enhancing ventral epidural abscess from C3-7. C: Intra-operative lateral plain x-ray films demonstrating C3-7 posterior decompression/fusion with C4-5 corpectomy and C3-6 anterior decompression/fusion.

67-year-old otherwise healthy female who presented to the emergency department with 3 weeks of neck pain and progressive mild weakness in her bilateral upper extremities. MRI with and without contrast was obtained in the emergency department which demonstrated C4-5 spondylodiscitis with developing kyphosis with associated C3-7 ventral epidural abscess (Fig. 1A, B). CRP was 4.08 mg/dL and ESR 129 mm/h. There was concern for potential tracheal compression from a prevertebral phlegmon, so blood cultures were drawn, and the patient was started on empiric broad spectrum antibiotics.

The patient was then brought to the operating room and underwent posterior C3-7 decompression and fusion with both allograft and autograft as well as C4-5 corpectomy with C3-6 anterior decompression and fusion with allograft (Fig. 1C). Intraoperative cultures were positive for Methicillin-resistant *Staphylococcus aureus*. The patient's antibiotic regiment was narrowed to intravenous daptomycin for 6 weeks with plans for potentially lifelong oral suppression antibiotics and her neurological exam has remained stable post-operatively.

##### 3.3.5.2 Case 2

**Fig. 2:**
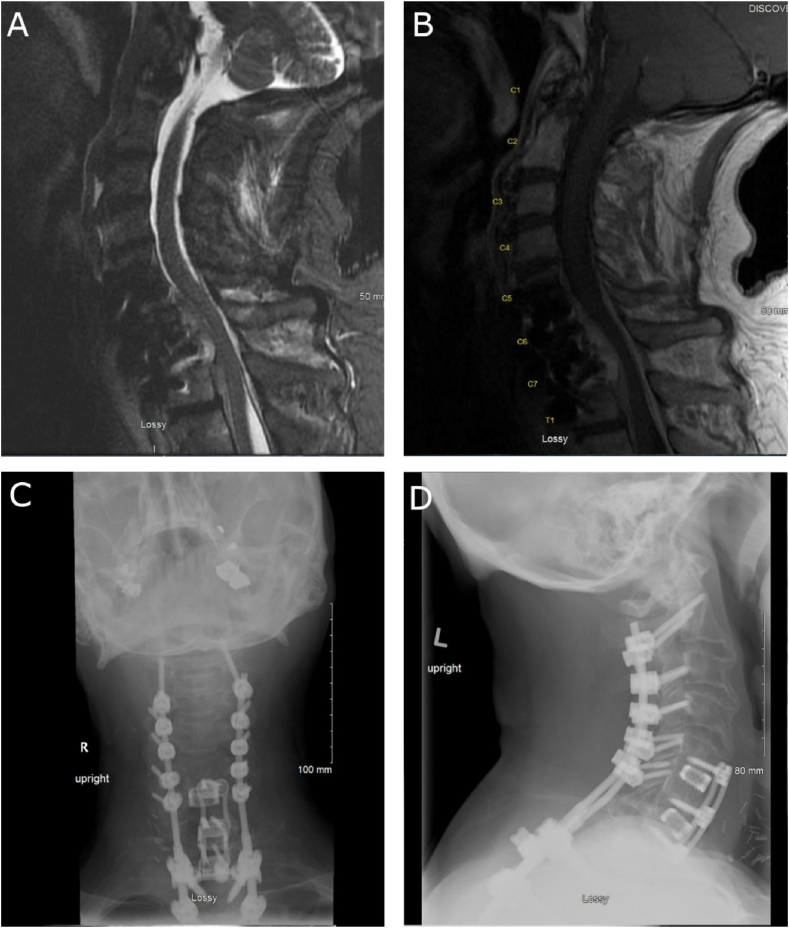
Example of a case of cervical spondylodiscitis with epidural abscess treated at our center. A: Sagittal STIR image demonstrates significant compression of the lower cervical spinal cord at the levels of C6-T1. Subtle increased intensity in the cord at this level. B: Sagittal T1 post-contrast image demonstrates significant contrast enhancement within the epidural space ventrally at the levels of C5-T1 as well as dorsally from C5-T2. There is also evidence of extension of the infection into the C5–C7 prior fusion. Focal kyphosis can also be seen at the level of C7/T1. C–D: Post-operative anterior-posterior (C) and lateral (D) plain x-ray films demonstrating posterior instrumentation from C2-T4 as well as stable anterior C5-7 prior instrumentation. Spinal alignment is improved compared to pre-op.

61-year-old male with numerous medical co-morbidities as well as previous C5-7 anterior cervical diskectomy and fusion performed a year prior at a different medical center who presented to neurosurgery clinic 4 months after he had a mediastinal and neck dissection for polymicrobial abscess (treated with piperacillin-tazobactam followed by levofloxacin suppression therapy) with severe neck pain and myelopathy. Repeat imaging was obtained and demonstrated significant both ventral (C5-T1) and dorsal epidural abscess (C5 to T2) with significant canal stenosis as well as new focal kyphosis centered at C7/T1 (Fig. 2A, B). Repeat CRP was elevated at 8.11 mg/dL and ESR was 95 mm/h. Given his history of previous anterior fusion as well as multiple neck explorations, we performed a laminectomy from C5 to T1 with evacuation of epidural abscess with instrumentation and fusion (utilizing autograft as well as left iliac crest autograft) from C2 to T4 and facetectomies at C7/T1 and T1/2 with correction of his post-infectious kyphosis (Fig. 2C, D). Intraoperative cultures positive for Methicillin-resistant *Staphylococcus aureus*, which was not found on initial intraoperative cultures.

The patient was continued on levofloxacin for his previous infection and started on IV vancomycin for a total of 7.5 weeks followed by doxycycline and continued levofloxacin suppressive therapy, which at this time has no specific end point. The patient had improvement in his inflammatory markers with marked improvement in his neck pain and gradual improvement in his neurological complaints.

## 4 Conclusion

**Fig. 3:**
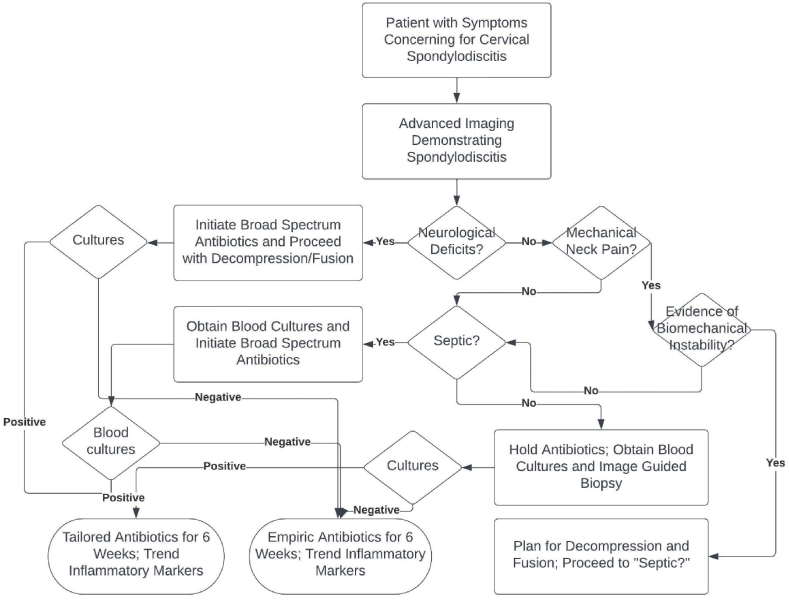
Flowsheet for initial treatment of patients presenting with spondylodiscitis.

Spondylodiscitis of the cervical spine is an increasingly more common and potentially devastating complication of the ever-increasing medical comorbidities of the current population. Accurate diagnosis and swift treatment can lead to good outcomes in most patients and can reduce the need for surgical intervention. When surgical intervention is required, particular attention should be given to stabilization of the cervical spine and decompression of the spinal cord or neuronal elements (particularly with an anterior approach when appropriate) with correction of associated deformity to offer the patient the best neurological outcome. Based on our review of the literature, we developed a simple treatment algorithm to assist in the initial treatment of patients with cervical spondylodiscitis (Fig. 3).

## Author contribution statement

All authors listed have significantly contributed to the development and the writing of this article.

## Funding

This research was not supported by funding.

## Data availability statement

No data was used for the research described in the article.

## Declaration of competing interest

The authors declare that they have no known competing financial interests or personal relationships that could have appeared to influence the work reported in this paper.

## Abbreviations

CRP: C-reactive protein
CT: computed tomography
ESR: Erythrocyte sedimentation rate
MRI: magnetic resonance imaging
PCT: procalcitonin
PEEK: polyetheretherketone
